# Comparison of high flow nasal cannula and non-invasive positive pressure ventilation in children with bronchiolitis: A meta-analysis of randomized controlled trials

**DOI:** 10.3389/fped.2022.947667

**Published:** 2022-07-15

**Authors:** Zhaoshuang Zhong, Long Zhao, Yan Zhao, Shuyue Xia

**Affiliations:** Department of Respiratory, Central Hospital, Shenyang Medical College, Shenyang, China

**Keywords:** high-flow nasal cannula, non-invasive positive pressure ventilation, bronchiolitis, children, CPAP (continuous positive air pressure)

## Abstract

**Background:**

The effects of high-flow nasal cannula (HFNC) compared to non-invasive positive pressure ventilation (NIPPV) on children with bronchiolitis remain unclear.

**Methods:**

This meta-analysis was performed following the preferred reporting items for systematic reviews and meta-analysis (PRISMA) statement. Randomized controlled trials (RCTs) were identified from a comprehensive search in PubMed, EMBASE, Cochrane Library, and Web of Science without time and language limitations. Primary endpoints include the rate of treatment failure, the rate of need for intubation, and the pediatric intensive care unit (PICU) length of stay.

**Results:**

Five RCTs including 541 children of less than 24 months were enrolled in the meta-analysis. Compared to the NIPPV group, the rate of treatment failure was significantly higher in the HFNC treatment group (*I*^2^ = 0.0%, *P* = 0.574; RR 1.523, 95% CI 1.205 to 1.924, *P* < 0.001). No significant difference was noted in the need for intubation (*I*^2^ = 0.0%, *P* = 0.431; RR 0.874, 95% CI 0.598 to 1.276, *P* = 0.485) and the PICU length of stay (*I*^2^ = 0.0%, *P* = 0.568; WMD = –0.097, 95% CI = –0.480 to 0.285, *P* = 0.618) between the HFNC group and the NIPPV treatment.

**Conclusion:**

Compared to the NIPPV group, HFNC therapy was associated with a significantly higher treatment failure rate in children suffering from bronchiolitis. The intubation rate and the PICU length of stay were comparable between the two approaches.

## Introduction

Bronchiolitis is an acute infection of the lower respiratory tract and one of the significant causes of illness and hospitalization in young children (1, 2). It is usually caused by the respiratory syncytial virus (RSV), which almost all children will be infected by 2 years of age (3, 4). Severe bronchiolitis is featured with airway obstruction, hypoxemia, increased work of breathing, and respiratory distress, which need advanced supportive management, including hydration, oxygen support, or assisted ventilation (5–7). Given the complications of mechanical ventilation, non-invasive positive pressure ventilation (NIPPV), such as continuous positive airway pressure (CPAP) and nasal positive pressure ventilation (NPPV), has been widely used and proved to be effective in the treatment of bronchiolitis (8–12).

The high-flow nasal cannula (HFNC) is another choice (13, 14). Compared to a simple nasal cannula, HFNC can reduce the dead space in the nasopharynx, decrease breathing work, and provide proper humidity and temperature (15, 16). Moreover, it is staff-friendly and more comfortable for children than CPAP since there is no need for close monitoring and a stressful tight-fitting interface (17, 18). Several trials and systematic reviews have compared the effects of HFNC and NIPPV; however, the results were inconsistent or inconclusive for the shortage of evidence. Therefore, to clarify this issue, we conducted this updated meta-analysis of HFNC versus NIPPV in infants with bronchiolitis.

## Methods

### Search strategy

This meta-analysis was performed following the preferred reporting items for systematic reviews and meta-analysis (PRISMA) statement (19). All published RCTs investigating the effects of HFNC compared with NIPPV (including CPAP and NPPV) were sought out by a comprehensive search in PubMed, EMBASE, Cochrane Library, and Web of Science from the establishment to May 2022 without language restriction. Search formula was performed as (high flow nasal cannula) AND (bronchiolitis) AND (children) AND (randomized). Two authors (ZZ and LZ) performed the selection independently and resolved disagreements by referring to the third author (SX).

### Inclusion and exclusion criteria

Trials were enrolled if they met the following inclusion criteria: (1) RCT; (2) HFNC treatment was applied and compared with the NIPPV method, and (3) reported at least one of the following outcomes: the rate of treatment failure (defined as the author of each trial), the rate of need for intubation, and the pediatric intensive care unit (PICU) length of stay. Duplicated literature, reviews, conference abstracts, and case reports were excluded.

### Data extraction and quality assessment

Two independent authors (ZZ and LZ) evaluated each trial’s eligibility and methodological quality according to the Modified Jadad scale (20). Data were extracted using a pre-designed structured form, including data elements: (1) general information, such as the name of the author, population size, year of publication, and study design; (2) patient characteristics, such as age, weight, the proportion of RSV positive, the baseline value of heart rate, respiratory rate, and SPO2; (3) outcomes as mentioned above. The disagreements were resolved by consensus or referring to the third author (SX).

### Statistical analysis

The pooled risk ratios (RRs) with 95% confidence intervals (CI) and the weighted mean difference (WMD) with 95% CI were calculated for dichotomous outcomes and continuous outcomes respectively. Inter-study heterogeneity was measured by the *I*^2^ test, and a random-effects (RE) model was used for all pooled outcomes (21). In the case of high heterogeneity, the sensitivity or subgroup analysis would be considered. Publication bias was evaluated using a funnel plot with Begg’s test (22). Results with a two-sided *P*-value < 0.05 indicated a statistical significance. All statistical analyses were performed using Stata v12.0 (Stata Corp., College Station, TX, United States) with the metan function.

## Results

### Search results and study characteristics

**FIGURE 1:**
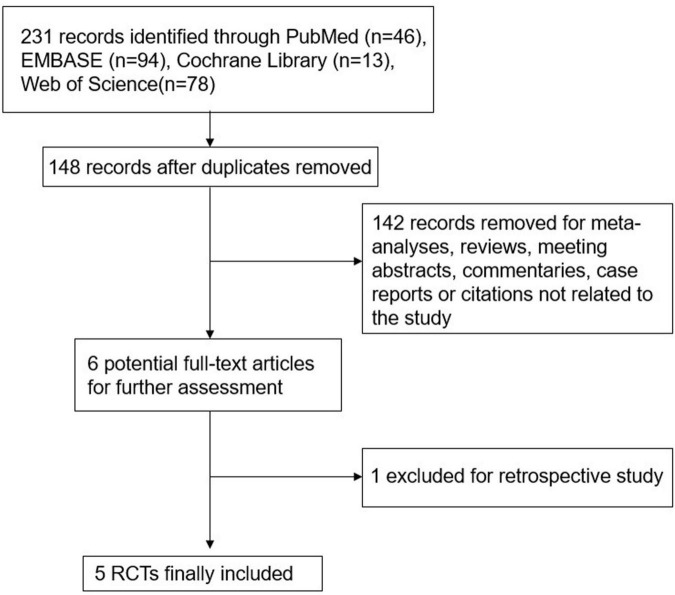
Flow chart of study selection.

A total of 231 titles and abstracts were identified by the search strategy, in which 83 duplicate records were excluded. Another 142 citations were removed as reviews, conference abstracts, case reports, or irrelevant studies by screening the titles and abstracts. In the six articles retrieved for full-text review, one was excluded for retrospective design. Eventually, five RCTs (23–27) involving a total of 541 children of less than 24 months were included in the present meta-analysis. The detailed literature selection process is illustrated in Figure 1. The baseline characteristics of the enrolled studies are presented in Table 1, and more detailed information (such as treatment failure criteria, timing of failure, the predictors of treatment failure, and the initial settings) is summarized in online Supplementary Table 1.

**TABLE 1:** Characteristics of included studies.

Study	Year	NIPPV strategy	NIPPV interface	HG/NG	Sample size, n	Age, m	Weight, kg	RSV positive, *n* (%)	RR	HR	SPO2(%)
Borgi et al. (23)	2021	CPAP/NPPV	Nasal mask or nasal prong	HG	130	1.8 ± 1.2	4.6 ± 1.3	46 (35.4)	68 ± 14.8	170.2 ± 16.5	90.3 ± 6.9
				NG	125	1.6 ± 1.1	4.4 ± 1.2	44 (35.2)	65.9 ± 14.5	170.4 ± 19.0	91.2 ± 6.8
Vahlkvist et al. (24)	2020	CPAP	Nasal prong	HG	22	2.1 (0.5–8.8)	5.2 (3.3–8.6)	20 (90)	56 ± 12	156 ± 21	NR
				NG	28	2.8 (0.3–11.3)	5.2 (2.8–9.7)	25 (89)	60 ± 15	155 ± 22	
Cesar et al. (25)	2020	CPAP	Nasal prong	HG	35	3.4 (1.4–5.4)	5.9 ± 1.8	30 (85.7)	49.2 ± 10.3	147.6 ± 22.6	97.5 (95–99)
				NG	28	2.4 (0.9–3.3)	5.5 ± 1.5	26 (92.9)	49.2 ± 10.7	152.4 ± 18.1	98 (96–99)
Sarkar et al. (26)	2018	CPAP	Nasal mask or nasal prong	HG	15	4.1 ± 2.9	NR	NR	73.6 ± 3.6	164 ± 8.8	88.3 ± 2.5
				NG	16	2.8 ± 1.0			72.8 ± 3.7	168.5 ± 5.7	88.8 ± 1.4
Milési et al. (27)	2017	CPAP	NR	HG	71	1.4 ± 1.3	4.1 ± 1.3	125 (88)	52 ± 18	166 ± 20	95 ± 5
				NG	71	1.3 ± 1.1	4.1 ± 1.1		54 ± 18	165 ± 19	95 ± 4

*HG high flow nasal cannula group, NG non-invasive positive pressure ventilation group, CPAP continuous positive airway pressure, NPPV nasal positive pressure ventilation, NIPPV non-invasive positive pressure ventilation, RSV respiratory syncytial virus, RR respiratory rate, HR heart rate, SPO2 oxygen saturation, NR not reported.*

### Quality assessment and publication bias

**FIGURE 2:**
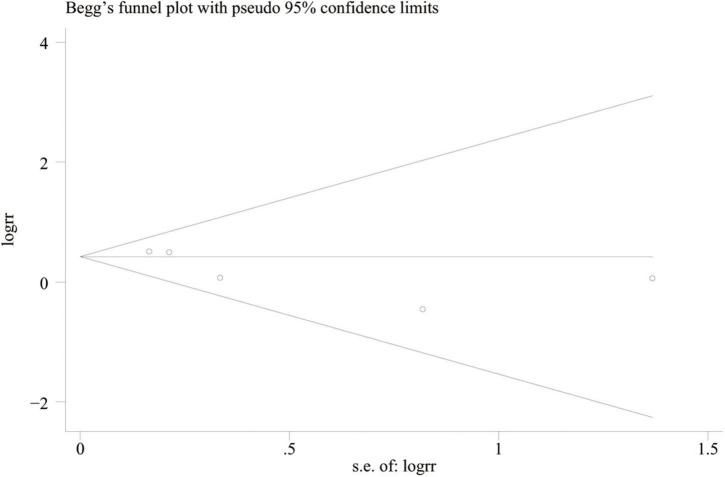
Funnel plot for treatment failure rate.

The methodological quality was evaluated by the Modified Jadad scale, including randomization, double-blinding, withdrawals and dropouts, and allocation concealment (20). The scores of the enrolled trials are summarized in Table 2, ranging from 4 to 5. No publication bias was found using a symmetrical funnel plot based on the outcome of treatment failure (Begg’s test, *P* = 0.462) (Figure 2).

**TABLE 2:** Assessment of methodological quality of included studies.

Author	Randomization	Double blinding	Allocation concealment	Withdrawals/dropouts	Scores
Borgi et al. (23)	Yes	No	Unclear	Yes	4
Vahlkvist et al. (24)	Yes	No	Yes	Yes	5
Cesar et al. (25)	Yes	No	Unclear	Yes	4
Sarkar et al. (26)	Yes	No	Unclear	Yes	4
Milési et al. (27)	Yes	No	Yes	Yes	5

### Meta-analysis results

#### Treatment failure

**FIGURE 3:**
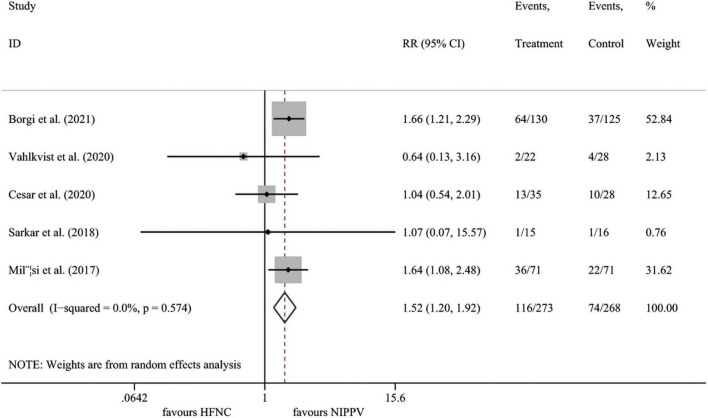
Forest plot for treatment failure rate. RR, relative risk; HFNC, high flow nasal cannula; NIPPV, non-invasive positive pressure ventilation.

All five studies (23–27) involving 541 cases reported the rate of treatment failure. Compared to the NIPPV group, the rate of treatment failure was significantly higher in the HFNC treatment group (*I*^2^ = 0.0%, *P* = 0.574; RR 1.523, 95% CI 1.205 to 1.924, *P* < 0.001) (Figure 3).

#### Need for intubation

**FIGURE 4:**
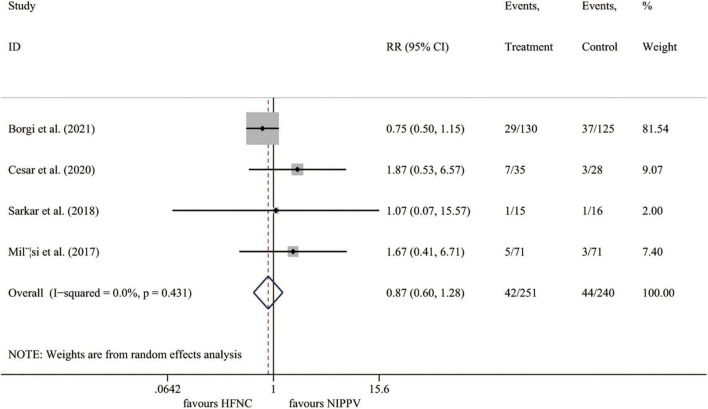
Forest plot for intubation rate. RR, relative risk; HFNC, high flow nasal cannula; NIPPV, non-invasive positive pressure ventilation.

Four trials (23, 25–27) reported the intubation rate, including 491 cases. Compared to the NIPPV group, the HFNC therapy showed no benefits on the incidence of intubation (*I*^2^ = 0.0%, *P* = 0.431; RR 0.874, 95% CI 0.598 to 1.276, *P* = 0.485) (Figure 4).

#### Pediatric intensive care unit length of stay

**FIGURE 5:**
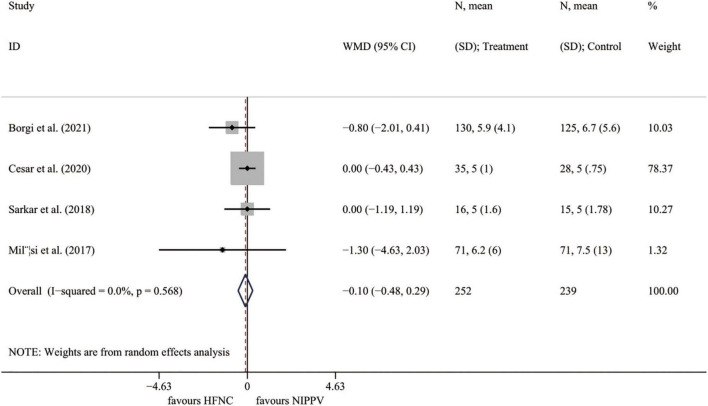
Forest plot for PICU length of stay. PICU, pediatric intensive care unit; WMD, weighted mean difference; HFNC, high flow nasal cannula; NIPPV, non-invasive positive pressure ventilation.

Four RCTs (23, 25–27) presented the outcome of PICU stay length. The results demonstrated no significant difference between the HFNC group and the NIPPV approach (*I*^2^ = 0.0%, *P* = 0.568; WMD = –0.097, 95% CI = –0.480 to 0.285, *P* = 0.618) (Figure 5).

## Discussion

In the present meta-analysis, we investigated the effects of HFNC compared to NIPPV in children with bronchiolitis. The main finding was that the rate of treatment failure was significantly higher in the HFNC group. For other outcomes, the rate of intubation and the PICU length of stay were comparable between the two groups.

In previous studies, HFNC was associated with a lower rate of treatment failure and intubation in young children with bronchiolitis (28, 29). Similarly, several studies investigated the effects of NIPPV in the treatment of bronchiolitis and proved that NIPPV, especially for CPAP, was superior to the standard treatment (8, 30). Compared to the NIPPV, HFNC was more comfortable and acceptable for the children and was associated with a lower rate of adverse events such as nasal injury (26, 27). However, though studies have compared the effects of HFNC and NIPPV, the results were inconsistent or inconclusive. Milesi et al. reported that in young infants with moderate to severe bronchiolitis, initial management with HFNC had a higher treatment failure rate than CPAP (27). On the contrary, Vahlkvist et al. (24) and Cesar et al. (25) found that treatment with HFNC led to a rate of treatment failure comparable to CPAP. Cataño-Jaramillo et al. conducted a systematic review including three RCTs to investigate this issue (31). Although they found a trend favoring CPAP over HFNC for the outcome of treatment failure, there was a lack of statistical significance (*P* = 0.05). Our study, including five RCTs and 541 cases, almost doubles the previous research, making the conclusion clearer than ever.

Non-invasive positive pressure ventilation was widely used in the treatment of respiratory illnesses. As investigated by the previous publications, the benefits might mainly be driven by providing positive end-expiratory pressure (PEEP), resulting in distending airway pressure on the distal airway. This effect may decrease the airways’ resistance and help prevent alveolar collapse and obstructive apnea (8). HFNC is suggested to deliver a warm and humidified gas and reduce the airway dead space and resistance (15, 32). As reported, HFNC may also generate a potential PEEP in the airways (33). Flow rates of ≥ 6 L/min appear to provide positive pressure throughout the respiratory cycle, with a PEEP range from 2 to 5 cm H_2_O (34, 35); however, the pressure is variable and unmonitored, depending on the weight of infants, flow rate, and leaks through the mouth and nares. All these factors may limit its effects in young children with bronchiolitis (14). In the present study, we did not notice any significant difference between the two approaches for the rate of intubation and PICU length of stay, which might be affected by the restricted study number and population, as well as the fact that for infants treated with HFNC a switch toward NIPPV group was allowed before intubation in some studies (23). Besides, most of the adverse effects were minor and comparable between the two groups.

As far as we know, the present study is the first meta-analysis to ascertain the superiority of NIPPV compared to HFNC on the primary outcome of treatment failure in children with bronchiolitis. However, the potential limitations of the study should not be ignored. First, though RCTs were enrolled, the total patient number of the study was only 541 cases, and the analysis power might be affected. Second, since the devices were clinically widely used and recognizable, none of the studies was blinded, which may lead to a performance bias. Similarly, only three trials reported the method of concealment, which means selection bias may affect the results. Third, because only five trials were included for assessment, the power of the funnel plot asymmetry test for publication bias might be restricted. Finally, the meta-analysis contained studies with inconsistent intervention regimens and children’s clinical features, which may also affect the results. For example, Borgi et al. included both CPAP and NPPV in the same group, and the success rate could have been influenced by using NPPV with variable pressure compared to CPAP with constant pressure. Therefore, the study results should be interpreted cautiously, and more RCTs with a larger population were expected.

## Conclusion

Compared to the NIPPV approach, HFNC therapy was associated with a significantly higher treatment failure rate in children suffering from bronchiolitis. The intubation rate and the PICU length of stay were comparable between the two groups. Given the study’s limitations, the results should be interpreted cautiously, and further investigation is warranted.

## Data Availability Statement

The original contributions presented in this study are included in the article/Supplementary Material, further inquiries can be directed to the corresponding author.

## Author contributions

ZZ and SX conceived and designed the study. ZZ, LZ, and SX performed the study and wrote the main manuscript text. LZ and YZ contributed analysis tools and prepared figures. All authors reviewed the manuscript.

conflict@pubnote

## Conflict of Interest

The authors declare that the research was conducted in the absence of any commercial or financial relationships that could be construed as a potential conflict of interest.

## Publisher’s Note

All claims expressed in this article are solely those of the authors and do not necessarily represent those of their affiliated organizations, or those of the publisher, the editors and the reviewers. Any product that may be evaluated in this article, or claim that may be made by its manufacturer, is not guaranteed or endorsed by the publisher.
