# Supplementary material for: Comparison of high flow nasal cannula and non-invasive positive pressure ventilation in children with bronchiolitis: A meta-analysis of randomized controlled trials
Source: Front Pediatr. 2022 Jul 15;10:947667. doi: 10.3389/fped.2022.947667 (PMC9334708; doi:10.3389/fped.2022.947667)
Supplement: Supplementary file 1 [file Table_1.DOCX]

**online suppl. Table 1 Detailed information of included studies**

| Study | Year | HG/NG | Treatment failure criteria | Timing of failure, hrs | Failure predictor | Initial settings |
| --- | --- | --- | --- | --- | --- | --- |
| Borgi et al.[23] | 2021 | HG | FiO_2_> 60% to maintain SpO_2_≤ 90% or increasing of work of breathing | NR | Younger age, lower weight, lower pH, and higher pCO_2_ | Flow rate was usually started at the maximum flow rate for the size of the cannula and a constant flow temperature of 37°C. The starting FiO_2_ was what could maintain SpO_2_ of 94%. |
|  |  | NG |  |  | Lower baseline pH | CPAP: starting pressure was 6cm H_2_O; maximum of 8cm H_2_O. NPPV: starting pressure was 20cm H_2_O with a maximum pressure at 30, maximum PEEP was 8cm H_2_O and maximum frequency was 35 cycles/min, inspiratory time was 0.7 seconds, and flow gas was 15L/min. |
| Vahlkvist et al.[24] | 2020 | HG | Switch of the two groups or transmission to the pediatric intensive care unit assessed by the physician | NR | NR | The initial flow was 2L/kg/min. Oxygen supply was delivered as needed to maintain a SpO_2_ above 92%. |
|  |  | NG |  |  |  | The initial flow was 12–14L/min. Oxygen supply was delivered as needed to maintain a SpO_2_ above 92%. |
| Cesar et al.[25] | 2020 | HG | Escalate support to noninvasive bi-level pressure ventilation, or endotracheal intubation | 15.2 (12.5–25) | No predictors were found in baseline characteristics | Flow was titrated up to a maximum of 1.5 L/kg/min. FiO_2_ was adjusted to achieve a SpO_2_ >93%. |
|  |  | NG |  | 18.8 (9.1–41) |  | Pressure was set at 6 cm H_2_O. FiO_2_ was adjusted to achieve a SpO_2_ >93%. |
| Sarkar et al.[26] | 2018 | HG | HR and/or RR remained unchanged/increased; required FiO_2_ >60% for CPAP with PEEP >8; required FiO_2_ >60% for HFNC with maximum O_2_ flow rate to maintain SpO_2_ >94% and no improvement or increase in RDAI score. | NR | NR | Flow rate of 2L/kg/min for the children less than equal to 10 kg and for children >10 kg 2L/kg/min for the first 10 kg + 0.5L/kg/min for each kg above that and FiO_2_ 0.4 at initiation. |
|  |  | NG |  |  |  | Pressure was started at 4 cm H_2_O and increased as necessary up to a maximum of 8 cm H_2_O. |
| Milési et al.[27] | 2017 | HG | Occurrence of one of the following criteria: (1) a 1-point increase in mWCAS compared with baseline; (2) RR rise >10 bpm compared with baseline, with RR >60 bpm; (3) a 1-point increase in the EDIN score compared with baseline, with EDIN >4; and (4) more than two severe apnea episodes per hour. | 9.7±8.8 | Higher baseline FiO_2_ | Flow was delivered at 2L/kg/min. FiO_2_ was titrated in order to achieve a SpO_2_ of 94–97%, and the humidifier was auto set at 37 °C. |
|  |  | NG |  | 6.7±5.7 | Higher weight | Pressure was set at +7 cmH_2_O. |

*HG* high flow nasal cannula group, *NG* noninvasive positive pressure ventilation group, *CPAP* continuous positive airway pressure, *NPPV* nasal positive pressure ventilation, *RR* respiratory rate, *HR* heart rate, *SPO_2_* oxygen saturation, *FiO_2_* fraction of inspired oxygen, *PEEP* positive end-expiratory pressure, *RDAI* respiratory distress assessment index, *mWCAS* modified Wood’s clinical asthma score, *EDIN* neonatal pain and discomfort scale, *NR* not reported.
